# Burden in caregivers of patients with schizophrenia, depression, dementia, and stroke in Japan: comparative analysis of quality of life, work productivity, and qualitative caregiving burden

**DOI:** 10.1186/s12888-024-06000-x

**Published:** 2024-09-02

**Authors:** Yoshitsugu Kojima, Sakiko Yamada, Kunitoshi Kamijima, Kentaro Kogushi, Shunya Ikeda

**Affiliations:** 1 Medical Affairs, Otsuka Pharmaceutical Co., Ltd. Shinagawa Grand Central Tower, 2-16-4 Konan, Minato-ku, Tokyo, 108-8242 Japan; 2 Showa University, Tokyo, Japan; 3 Department of Public Health, School of Medicine, International University of Health and Welfare, Narita, Japan

**Keywords:** Caregivers, Burden, Schizophrenia, Alzheimer’s disease/dementia, Stroke, Depression, Quality of life, Work productivity, Cost

## Abstract

**Background:**

The main objective of this study was to examine the burden of schizophrenia, depression, Alzheimer’s disease/dementia, and stroke on caregivers and non-caregivers in Japan. This study also aimed to provide a comparative landscape on the burden of caregiving for each disorder.

**Methods:**

The Japan National Health and Wellness Survey database, 2016 and 2018 was used in this study. Health-related quality of life (HRQoL), work productivity, and health care utilization were assessed using a self-administered, Internet-based questionnaire. The burden of caregiving experienced by each group of caregivers was compared with background-matched non-caregivers (controls) as well as with caregivers of patients with each disorder.

**Results:**

Caregivers of patients with schizophrenia, depression, Alzheimer’s disease/dementia, or stroke had lower HRQoL, higher healthcare costs and work productivity impairment than non-caregivers. Furthermore, caregivers of patients with psychiatric disorders such as schizophrenia and depression had lower HRQoL and work productivity than caregivers of patients with Alzheimer’s disease/dementia and stroke. In addition, according to the Caregiver Reaction Assessment (CRA), caregivers of patients with schizophrenia and depression were more inclined to perceive a loss in physical strength and financial burden to the same extent as their self-esteem.

**Conclusions:**

This study indicated a substantial caregiving burden among caregivers of patients with psychiatric and neurological diseases in Japan. The caregiver burden of psychiatric disorders (schizophrenia and depression) was greater than that of neurological disorders (Alzheimer’s disease/dementia and stroke), suggesting a need to provide support to caregivers of patients with psychiatric disorders to be better able to care for their patients.

**Trial Registration:**

None.

**Supplementary Information:**

The online version contains supplementary material available at 10.1186/s12888-024-06000-x.

## Background

The number of patients with mental disorders is increasing in Japan as well as worldwide. In fact, in Japan, the number of patients with mental disorders, including outpatients and inpatients, increased from 2.584 million in 2006 to 3.924 million in 2018, approximately 1.5 times [1]. Caregivers for patients with mental disorders experience a wide range of burdens, not only financially and physically but also psychologically. The economic loss from the social activities of family caregivers is estimated at over 500 billion USD per year in the United States (US) [2]. Moreover, in Japan, government care insurance policies have shifted from the initial focus of hospital-based care toward community-based care [3], leading to increased community care and caregiver burden. The social financial burden caused by long-term care expenditure is remarkable in Japan, which is more than 100 billion USD [3].

Dementia is one of the leading causes of disability and dependency among the elderly worldwide. It has physical, psychological, social, and economic consequences not only for patients but also for caregivers, family members, and society at large [4]. Alzheimer’s disease is known to be the most common type of dementia, accounting for 60–70% of all cases of dementia [4], and is estimated to affect at least 131.5 million people worldwide by 2050 [5]. Patients with dementia often suffer from physical, mental, and cognitive decline, contributing to an increased burden of care. A Swedish study showed that dementia increased the burden of caregiving, with an increase in caregiving time as cognitive function declined [6]. The results of the meta-analysis suggested that the difference in burden was large between caregivers and non-caregivers, with caregivers having lower psychological and physical health and subjective well-being than non-caregivers [7].

Stroke is the major cause of death [8], and even if a patient survives, it is often accompanied by serious, long-term disability [9–11]. Stroke onset is an unexpected event wherein patients are often hospitalized for shorter periods. This creates additional caregiver roles and an abrupt transition of lifestyle for the family [10, 12]. Caregivers of patients with stroke reportedly also have poorer physical and mental health with symptoms of anxiety and depression [13, 14]. Furthermore, the informal care of stroke survivors is associated with humanistic burden, including reduced health-related quality of life (HRQoL) and increased indirect economic costs, such as limited work productivity [15].

The course of schizophrenia is generally chronic, with acute psychotic relapses causing cognitive impairment [16, 17]. Schizophrenia causes impaired social functioning, which makes it difficult to work, and more than 60% of patients experience significantly impaired social functioning [18]. Additionally, patients with prominent symptoms of schizophrenia were reported to be more burdensome for their caregivers [19].

Depression is a common mental disorder that causes not only decreased activity but also functional impairment and is the most disabling disorder worldwide in terms of years lived with disability (YLDs) [20]. Recent reports indicate that cognitive decline persists even after a depressive episode has resolved [21]. Caring for depressed patients not only increases the burden of care but also raises the issue of “caregiver depression,” in which family caregivers experience high levels of depression, stress, and anxiety [22].

Similar to Alzheimer’s disease/dementia and stroke, psychiatric disorders (schizophrenia and major depressive disorder) are accompanied by a functional decline of patients [21] and an increased burden on caregivers [23]. Furthermore, it has been noted that the burden of care differs between patients with schizophrenia and those with chronic neurological disorders [24]. Although schizophrenia is associated with a lower objective burden of care than neurological disorders, the subjective burden is greater. This may be due to stigma, low social acceptability, or inadequate social support in individuals with psychiatric disorders [25]. While previous studies have suggested an increased burden of caregiving for patients with Alzheimer’s disease and schizophrenia compared to non-caregivers and caregivers of patients with other conditions [26–28], studies that quantitatively or qualitatively compare the burden are limited, especially among caregivers of patients with mental disorders.

In Japan, dementia (24.8%) and stroke (18.4%) have been reported as the most common factors requiring long-term care [29]. However, there are few studies on the burden of Japanese caregivers of patients with either schizophrenia or depression. Further, as the different health conditions require different types of care, which may impact caregiver burden, it is not known for the specific demands of the respective conditions may differentially impact caregivers. Therefore, the main purpose of this study was to investigate the HRQoL, work productivity, healthcare resource utilization, economic burden, and comorbid burden of caregivers of patients with schizophrenia, depression, Alzheimer’s disease/dementia, and stroke compared with those of caregivers in non-caregivers in Japan. This study also sought to provide a comparative landscape of the burden experienced among caregivers of patients with schizophrenia, depression, Alzheimer’s disease/dementia, or stroke in Japan.

## Methods

### Data sources and study design

This cross-sectional study used existing data from the 2016 and 2018 Japan National Health and Wellness Survey (NHWS). The Oracle Life Sciences (formerly known as Cerner Enviza) NHWS is a self-administered, Internet-based questionnaire administered to a nationwide sample of adults (aged 18 years or older). Potential respondents for this study were recruited through the general panel of Lightspeed Research (LSR). Panel members explicitly agreed to join the LSR panel and receive periodic invitations to participate in online surveys (i.e., not health-specific).

The survey received Institutional Review Board approval, and all the respondents provided informed consent before participating.

### Study sample

The Japan NHWS Survey database 2016 and 2018 were used for this analysis. If a respondent answered both the 2016 and the 2018 NHWS, only responses to the more recent NHWS 2018 were included in the study. Caregivers who cared for patients with a single relevant disease were included in the analysis, while caregivers who cared for multiple patients/patients with multiple diseases were excluded. Respondents (aged *≥* 18 years) who self-reported caring for an adult with Alzheimer’s disease, dementia, depression, schizophrenia, or stroke were included in the study. Caregivers of patients with Alzheimer’s disease and caregivers of patients with dementia were combined into a single group in the analysis. Propensity score matching with a greedy matching algorithm was performed by matching one caregiver of patients to one non-caregiver (1:1 matching). A total of 2,208 non-caregivers were expected to constitute the control group.

### Baseline and outcome measures

Baseline variables of the caregivers and non-caregivers assessed were the following: demographic factors (age, sex, marital status, education, household income, and employment status) and general health characteristics (body mass index [BMI], smoking status, alcohol consumption, exercise behavior in the past 30 days, and comorbidity score). The comorbidity score was calculated based on the Charlson Comorbidity Index (CCI) [30–32].

### Health outcomes

Health-related quality of life was assessed using the physical component summary (PCS) and mental component summary (MCS) from the 12-item Short-Form Health Survey version 2 (SF-12v2) [33]. The Short Form Six Dimension (SF-6D) index utilizes data from the SF-12v2 to calculate preference-based health utility index to understand the overall health state. The SF-6D is scored from 0.0 (worst health state) to 1.0 (best health state). Health state utilities were quantified by the Five Level EuroQol Five Dimension (EQ-5D-5 L) instrument, which is a standardized measure of health status to provide a simple, generic measure of health [34].

Work productivity and activity impairment were assessed using the Work Productivity and Activity Impairment (WPAI) questionnaire, a 6-item validated instrument consisting of four metrics: absenteeism (percentage of work time missed due to one’s health in the past seven days), presenteeism (percentage of impairment experienced while at work in the past seven days because of one’s health), overall work productivity loss (an overall impairment estimate that is a combination of absenteeism and presenteeism), and activity impairment (percentage of impairment in daily activities because of one’s health in the past seven days) [35].

Healthcare resource utilization was assessed by a few different items. Specifically, the number of traditional healthcare provider visits, the number of emergency room (ER) visits, and the number of times the patient was hospitalized in the past six months were reported.

The presence of depression was assessed by the validated Patient Health Questionnaire-9 (PHQ-9), a 9-item questionnaire measuring the frequency of depressive symptoms, with items scored on a 4-point scale (not at all = 0 to nearly every day = 3) [36]. The standard cut-off score for screening for identifying possible major depression is 10 or above [37].

The Caregiver Reaction Assessment (CRA) is a 24-item scale corresponding to the theoretical constructs of the Labor of Caregiving by measuring the impact of “taking care” related to managing the environment, preparing for death, and knowing one’s strengths [38]. Responses were measured on a 5-point Likert scale (strongly disagree = 1 to strongly agree = 5). The individual items were summed and averaged for a total score or a total subscale (impact on health, caregiver’s esteem, impact on schedule, impact on finances and lack of family support) score.

### Costs

Direct cost was estimated by multiplying the number of physician visits, ER visits, and hospitalizations by the corresponding unit cost for each component. The direct cost was quantified as an annual cost.

Indirect cost was calculated by using age- and sex-stratified wage information from Japanese yen [39] for each respondent multiplied by absenteeism, presenteeism, and overall work productivity loss. The indirect cost was quantified as annual absenteeism cost, annual presenteeism cost, and annual indirect cost.

### Statistical analysis

#### Descriptive analysis

The underlying distributions of sociodemographic factors, general health characteristics, and health outcomes for the included respondents were summarized to inform the appropriateness of the planned approach and the levels of the grouped variables. All variables were reported using counts, percentages, means, medians, interquartile ranges (IQRs), ranges, and/or standard deviations depending on the scale (nominal, ordinal, or continuous) of the item/measure.

#### Bivariate analysis

Differences between cases and controls (e.g., schizophrenia caregivers vs. non-caregivers, depression caregivers vs. non-caregivers, Alzheimer’s disease/dementia caregivers vs. non-caregivers, and stroke caregivers vs. non-caregivers) concerning the demographics and general health characteristics were first compared. Pearson’s chi-square test was used for categorical variables, and one-way analysis of variance (ANOVA) was used for continuous variables.

All covariates were used in the propensity score 1:1 matching process to create a control group of non-caregivers. Propensity score matching was carried out for caregivers of each condition separately to account for the potential differences between caregivers of different conditions. The R package MatchIt [40] was used for propensity score matching, with the “nearest” matching method and no reuse of controls. Post-matching bivariate analyses were repeated to determine whether any potentially confounding variables remained significantly unbalanced between the two groups. Standardized mean differences (SMDs) were used to assess the balance of matching. Variables with SMD greater than 0.10 were considered unbalanced after matching.

#### Analysis of primary objectives

Following propensity score matching, outcomes were compared between caregivers providing care to different conditions and their respective matched non-caregivers. Pearson’s chi-square test was used for comparing categorical variables and one-way ANOVA was used for comparing continuous variables. Bivariate comparisons were also conducted to compare the outcomes between caregivers of each condition and the outcomes of the combined matched non-caregiver controls.

#### Analysis of secondary objectives

To compare the burden among caregivers of different conditions, outcomes were compared among caregiver groups (pairwise comparison). Pearson’s chi-square test was used for comparing categorical variables and one-way ANOVA was used to compare continuous variables.

#### Multivariable analysis

In case of imbalance in matching between respective non-caregivers and caregivers of any conditions, multivariable analyses were used to evaluate the outcomes of these two groups. Caregiver status was used as the primary nominal predictor of health outcomes using generalized linear models (GLMs) to adjust for all covariates. GLMs with normal distribution and identity link functions were used for predicting normally distributed outcomes, such as HRQoL scores. GLMs with negative binomial distributions and log link functions were used for predicting outcome variables with skewed distributions, such as WPAI, number of physician visits, and costs. GLMs with binomial distributions and logit link functions were used for predicting outcome variables with binominal distributions, such as the PHQ-9, using a single cutoff of 10, with/without ER visits and with/without hospitalizations.

#### Sensitivity analysis

Propensity score 1:2 matching using a greedy matching algorithm was also carried out for caregivers of each condition separately for sensitivity analysis.

## Results

### Demographics of caregivers and non-caregivers

The demographic data of the caregivers and non-caregivers is shown in Table 1. From the Japan NHWS database 2016 (*n* = 39,000) and 2018 (*n* = 30,000), a total of 126 caregivers of patients with schizophrenia, 146 caregivers of patients with depression, 1,594 caregivers of patients with Alzheimer’s disease/dementia, and 342 caregivers of patients with stroke were included in the analyses and were compared to 47,909 non-caregivers. On average, caregivers of patients with Alzheimer’s disease/dementia were the oldest (55.1 years old), and caregivers of patients with depression were the youngest (45.5 years old). The proportion of caregivers who completed university education was the highest among caregivers of patients with Alzheimer’s disease/dementia (49.2%) and the lowest among caregivers of patients with schizophrenia (32.5%). Among caregivers of patients with depression, 67.8% were currently employed. Caregivers of patients with schizophrenia showed a higher proportion of underweight (BMI < 18.5) compared to other caregivers. One-quarter of the caregivers of patients with depression were current smokers, and the percentage of caregivers of patients with depression was the highest. More than 40% and 24.5% of caregivers of patients with Alzheimer’s disease/dementia, respectively, consumed alcohol at least 2 to 3 times per week and did at least 12 times of vigorous exercise in the past 30 days.

**Table 1:** Demographics and clinical characteristics

Non-caregiver/Caregiver	Non-caregivers (*N* = 47909)	Caregiver of Schizophrenia patients (*N* = 126)	Caregiver of Depression patients (*N* = 146)	Caregiver of Alzheimer’s disease/ dementia (*N* = 1594)	Caregiver of Stroke patients (*N* = 342)
	Mean ± SD, Median [IQR] or *n* (%)^a^	Mean ± SD, Median [IQR] or *n* (%)^a^	Mean ± SD, Median [IQR] or *n* (%)^a^	Mean ± SD, Median [IQR] or *n* (%)^a^	Mean ± SD, Median [IQR] or *n* (%)^a^
Age	51.66 ± 16.61	51.75 ± 16.83	45.45 ± 17.01	55.05 ± 15.02	53.19 ± 15.82
Charlson Comorbidity Index	0.0 [0.0; 15.0]	0.0 [0.0; 14.0]	0.0 [0.0; 4.0]	0.0 [0.0; 10.0]	0.0 [0.0; 7.0]
Gender					
Male	25,321(52.9)	60 (47.6)	68 (46.6)	799 (50.1)	154 (45.0)
Female	22,588 (47.1)	66 (52.4)	78 (53.4)	795 (49.9)	188 (55.0)
Marital Status					
Married or living with partner	30,265 (63.2)	71 (56.3)	84 (57.5)	1109 (69.6)	218 (63.7)
Not Married	17,559 (36.7)	55 (43.7)	61 (41.8)	485 (30.4)	124 (36.3)
Decline to answer	85 (0.2)	0 (0.0)	1 (0.7)	0 (0.0)	0 (0.0)
Level of Education					
Completed university education	22,119 (46.2)	41 (32.5)	68 (46.6)	785 (49.2)	136 (39.8)
Not	25,272 (52.8)	84 (66.7)	73 (50.0)	798 (50.1)	205 (59.9)
Decline to answer	518 (1.1)	1 (0.8)	5 (3.4)	11 (0.7)	1 (0.3)
Household Income					
< ¥3,000,000	8776 (18.3)	32 (25.4)	34 (23.3)	237 (14.9)	79 (23.1)
¥3,000,000 to < ¥5,000,000	12,017 (25.1)	37 (29.4)	35 (24.0)	386 (24.2)	95 (27.8)
¥5,000,000 to < ¥8,000,000	11,754 (24.5)	29 (23.0)	38 (26.0)	394 (24.7)	60 (17.5)
¥8,000,000 or more	9441 (19.7)	18 (14.3)	28 (19.2)	408 (25.6)	74 (21.6)
Decline to answer	5921 (12.4)	10 (7.9)	11 (7.5)	169 (10.6)	34 (9.9)
Employment Status					
Currently employed	27,666 (57.7)	72 (57.1)	99 (67.8)	893 (56.0)	192 (56.1)
Not	20,243 (42.3)	54 (42.9)	47 (32.2)	701 (44.0)	150 (43.9)
Body Mass Index					
Underweight (BMI < 18.5)	5159 (10.8)	23 (18.3)	12 (8.2)	146 (9.2)	31 (9.1)
Normal (BMI > = 18.5 & <25)	32,581 (68.0)	68 (54.0)	97 (66.4)	1076 (67.5)	223 (65.2)
Obese (BMI > = 25)	8615 (18.0)	26 (20.6)	32 (21.9)	333 (20.9)	76 (22.2)
Decline to answer	1554 (3.2)	9 (7.1)	5 (3.4)	39 (2.4)	12 (3.5)
Smoking Status					
Never	27,447 (57.3)	62 (49.2)	77 (52.7)	836 (52.4)	196 (57.3)
Former	11,800 (24.6)	39 (31.0)	34 (23.3)	441 (27.7)	80 (23.4)
Current	8662 (18.1)	25 (19.8)	35 (24.0)	317 (19.9)	66 (19.3)
Alcohol Use					
≤ once per week	29,719 (62.0)	82 (65.1)	88 (60.3)	913 (57.3)	220 (64.3)
≥ 2–3 times per week	18,190 (38.0)	44 (34.9)	58 (39.7)	681 (42.7)	122 (35.7)
Vigorous Exercise in Past 30 Days					
0–11 times	38,779 (80.9)	100 (79.4)	113 (77.4)	1204 (75.5)	291 (85.1)
≥ 12 times	9130 (19.1)	26 (20.6)	33 (22.6)	390 (24.5)	51 (14.9)

^a^ Mean ± SD: mean ± standard deviation; median [IQR]: median [interquartile range]; n (%): effective (percentage)

### Comparison between caregivers and non-caregivers

#### Schizophrenia

The demographic data of the caregivers and non-caregivers after 1:1 propensity score matching is shown in Table S1.

Most variables in baseline demographics were balanced, except for the level of education (SMD: 0.156) and household income (SMD: 0.128), between the caregivers of patients with schizophrenia and non-caregivers.

After propensity score matching, HRQoL, WPAI, healthcare resource utilization (HRU) and costs, PHQ-9 were compared between caregivers and their respective matched non-caregivers (Table 2).

**Table 2:** Outcome variables between caregivers of schizophrenia and non-caregivers after propensity score matching 1:1

	Non-caregiver		Caregiver of Schizophrenia Patients		
Continuous Variable	*N*	Mean ± SD [Median]	*N*	Mean ± SD [Median]	*p*-value^a^
Health-related Quality of Life					
Mental Component Summary (MCS)	126	46.71 ± 10.30 [46.83]	126	44.05 ± 11.27 [46.03]	0.051
Physical Component Summary (PCS)	126	51.98 ± 7.20 [53.85]	126	49.18 ± 8.50 [51.87]	0.005
EQ-5D	126	0.84 ± 0.17 [0.83]	126	0.78 ± 0.16 [0.78]	0.006
SF-6D	126	0.74 ± 0.13 [0.72]	126	0.70 ± 0.13 [0.69]	0.011
Work Productivity and Activity Impairment					
Absenteeism	35	4.51 ± 18.65 [0.00]	30	9.60 ± 19.51 [0.00]	0.287
Presenteeism	38	23.16 ± 26.52 [10.00]	31	37.10 ± 33.59 [40.00]	0.058
Total Work Productivity Impairment	34	20.62 ± 26.67 [10.00]	30	39.13 ± 35.63 [35.00]	0.021
Total Activity Impairment	126	22.62 ± 26.14 [20.00]	126	33.97 ± 28.98 [30.00]	0.001
Healthcare Resource Utilization					
No. of Physician Visits in the Past 6 Months	126	6.06 ± 10.85 [2.00]	126	8.44 ± 8.87 [6.00]	0.057
No. of ER Visits in the Past 6 Months	126	0.02 ± 0.13 [0.00]	126	0.17 ± 0.73 [0.00]	0.024
No. of Hospitalizations in the Past 6 Months	126	0.17 ± 1.10 [0.00]	126	0.50 ± 2.30 [0.00]	0.153
Cost					
Absenteeism Cost (thousand yen)	35	127.87 ± 532.32 [0.00]	30	338.28 ± 620.23 [0.00]	0.146
Presenteeism Cost (thousand yen)	38	791.22 ± 910.49 [470.13]	31	1397.45 ± 1365.62 [984.69]	0.031
Indirect Cost (thousand yen)	34	679.17 ± 873.60 [412.19]	30	1460.34 ± 1433.29 [984.13]	0.010
Direct Cost (thousand yen)	126	483.94 ± 2441.34 [34.68]	126	1239.48 ± 5024.51 [104.03]	0.130

Categorical Variable	N	%	N	%	p-value^b^
PHQ-9					
PHQ-9 Score < 10	106	84.1%	107	84.9%	0.862
PHQ-9 Score ≥ 10	20	15.9%	19	15.1%	

a: p-value based on one-way ANOVA

b: p-value based on Pearson’s chi-square test

In terms of HRQoL, caregivers of patients with schizophrenia had significantly lower PCS, EQ-5D index, and SF-6D compared to the non-caregivers (*p* < 0.05), however, the difference in MCS was not statistically significant (*p* = 0.051). Caregivers of patients with schizophrenia had significantly greater total work productivity and total activity impairment. Although caregivers of patients with schizophrenia had much higher means in absenteeism and presenteeism than non-caregivers, the differences were not statistically significant compared to non-caregivers. They had significantly more ER visits in the past 6 months, higher presenteeism cost, and indirect cost (*p* < 0.05). No difference was identified in PHQ-9 between caregivers of patients with schizophrenia and non-caregivers.

In the sensitivity analysis using 1:2 propensity matching, most of the key results were consistent with the 1:1 propensity matching results (Table 2). Caregivers of patients with schizophrenia showed significantly lower in all aspects of HRQoL, and greater presenteeism, total work productivity, and total activity impairment compared to non-caregivers (*p* < 0.05). Caregivers of patients with schizophrenia had significantly more physician visits, ER visits in the past 6 months, higher presenteeism cost, and indirect cost (*p* < 0.05) (data not shown).

Multivariable analyses adjusting for covariates were also conducted to evaluate the outcomes of these two groups. As shown in Table S2, after adjusting for potential confounding effects of demographics and clinical characteristic variables, caregivers of patients with schizophrenia scored significantly lower in MCS, PCS, EQ-5D, and SF-6D index compared to non-caregivers (*p* < 0.05). In addition, they had significantly greater total activity impairment, more healthcare utilization, higher indirect cost, and direct cost (*p* < 0.05).

#### Depression

The demographic data of the caregivers and non-caregivers after 1:1 propensity score matching are shown in Table S3.

After 1:1 propensity score matching, three variables, marital status (SMD: 0.168), household income (SMD: 0.142) and alcohol use (SMD: 0.128), were not balanced between caregivers of patients with depression and non-caregivers.

After propensity score matching, outcome variables were compared between caregivers and their respective matched non-caregivers (Table 3). Caregivers of patients with depression had significantly lower MCS, PCS, EQ-5D index, and SF-6D, compared to non-caregivers (*p* < 0.01). They had greater absenteeism, presenteeism, total work productivity impairment, and total activity impairment than non-caregivers (*p* < 0.05). They had significantly more physician visits and more hospitalizations in the past 6 months compared to non-caregivers (*p* < 0.05), however, the difference in the number of ER visits in the past 6 months was not statistically significant. In addition, they had significantly higher presenteeism cost, indirect cost, and direct cost (*p* < 0.05). No significant difference was identified in PHQ-9 between caregivers of patients with depression and non-caregivers.

**Table 3:** Outcome variables between caregivers of depression and non-caregivers after propensity score matching 1:1

	Non-caregiver		Caregiver of Depression Patients		
Continuous Variable	*N*	Mean ± SD [Median]	*N*	Mean ± SD [Median]	*p*-value^a^
Health-related Quality of Life					
Mental Component Summary (MCS)	146	48.27 ± 10.43 [50.51]	146	41.51 ± 10.68 [42.34]	< 0.001
Physical Component Summary (PCS)	146	51.94 ± 6.64 [53.35]	146	49.55 ± 7.21 [50.35]	0.003
EQ-5D	146	0.87 ± 0.13 [0.83]	146	0.74 ± 0.19 [0.74]	< 0.001
SF-6D	146	0.76 ± 0.13 [0.75]	146	0.67 ± 0.12 [0.65]	< 0.001
Work Productivity and Activity Impairment					
Absenteeism	54	3.89 ± 12.92 [0.00]	54	10.72 ± 21.21 [0.00]	0.046
Presenteeism	58	13.62 ± 19.80 [5.00]	59	28.14 ± 25.43 [20.00]	0.001
Total Work Productivity Impairment	54	16.37 ± 23.62 [10.00]	54	34.81 ± 30.30 [24.50]	< 0.001
Total Activity Impairment	146	19.59 ± 26.13 [10.00]	146	34.32 ± 27.09 [30.00]	< 0.001
Healthcare Resource Utilization					
No. of Physician Visits in the Past 6 Months	146	4.89 ± 6.32 [3.00]	146	8.01 ± 10.14 [6.00]	0.002
No. of ER Visits in the Past 6 Months	146	0.55 ± 6.13 [0.00]	146	0.64 ± 3.43 [0.00]	0.888
No. of Hospitalizations in the Past 6 Months	146	0.09 ± 0.52 [0.00]	146	0.84 ± 4.48 [0.00]	0.047
Cost					
Absenteeism Cost (thousand yen)	54	152.10 ± 525.40 [0.00]	54	430.77 ± 926.40 [0.00]	0.057
Presenteeism Cost (thousand yen)	58	511.00 ± 834.09 [137.76]	59	1005.20 ± 985.52 [608.64]	0.004
Indirect Cost (thousand yen)	54	615.02 ± 987.57 [276.64]	54	1261.24 ± 1231.66 [944.77]	0.003
Direct Cost (thousand yen)	146	313.64 ± 1211.32 [52.01]	146	1988.94 ± 9901.81 [121.37]	0.043

Categorical Variable	N	%	N	%	p-value^b^
PHQ-9					
PHQ-9 Score < 10	134	91.8%	124	84.9%	0.068
PHQ-9 Score ≥ 10	12	8.2%	22	15.1%	

a: p-value based on one-way ANOVA

b: p-value based on Pearson’s chi-square test

In the sensitivity analysis using 1:2 propensity matching, most of the key results were consistent with the 1:1 propensity matching results (Table 3). Caregivers of depressed patients had significantly higher absenteeism cost, indirect cost, and direct cost than non-caregiver (*p* < 0.05), however, the presenteeism cost was not statistically significant (data not shown).

Multivariable analyses adjusting for covariates were also conducted to evaluate the outcomes of these two groups. As shown in Table S4, after adjusting for potential confounding effects of demographics and clinical characteristic variables, caregivers of patients with depression scored significantly lower in MCS, PCS, EQ-5D, and SF-6D index compared to non-caregivers (*p* < 0.01). They had significantly higher presenteeism, impairment of total work productivity and total activity (*p* < 0.001), and more physician visits in the past 6 months (*p* < 0.001) and were more likely to be hospitalized in the past 6 months (*p* < 0.05). Also, they had higher presenteeism cost, indirect cost, and direct cost than non-caregivers (*p* < 0.01).

#### Alzheimer’s disease/dementia

The demographic data of the caregivers and non-caregivers after 1:1 propensity score matching is shown in Table S5.

Most variables were balanced except for BMI (SMD = 0.118) between caregivers of patients with Alzheimer’s disease/dementia and non-caregivers.

After propensity score matching, outcome variables were compared between caregivers and their respective matched non-caregivers (Table 4). Caregivers of patients with Alzheimer’s disease/dementia patients had significantly lower MCS, PCS, EQ-5D index, and SF-6D compared to non-caregivers (*p* < 0.001). They had greater absenteeism, presenteeism, total work productivity impairment, and total activity impairment, compared to non-caregivers (p *≤* 0.001). They had significantly more physician visits in the past 6 months compared to non-caregivers (*p* < 0.001), however, the differences in the number of ER visits and hospitalizations in the past 6 months were not statistically significant. In addition, they had significantly higher absenteeism cost, presenteeism cost, and indirect cost than non-caregivers (*p* < 0.01). A significantly higher proportion of caregivers of patients with Alzheimer’s disease/dementia had PHQ-9 score ≥ 10 (*p* = 0.003).

**Table 4:** Outcome variables between caregivers of Alzheimer’s disease/dementia and non-caregivers after propensity score matching 1:1

	Non-caregiver		Caregiver of Alzheimer’s disease/dementia Patients		
Continuous Variable	*N*	Mean ± SD [Median]	*N*	Mean ± SD [Median]	*p*-value^a^
Health-related Quality of Life					
Mental Component Summary (MCS)	1594	49.38 ± 9.32 [51.25]	1594	46.94 ± 10.37 [48.96]	< 0.001
Physical Component Summary (PCS)	1594	52.31 ± 5.89 [53.66]	1594	51.52 ± 6.24 [52.77]	< 0.001
EQ-5D	1594	0.87 ± 0.15 [0.83]	1594	0.82 ± 0.16 [0.81]	< 0.001
SF-6D	1594	0.77 ± 0.12 [0.79]	1594	0.73 ± 0.13 [0.72]	< 0.001
Work Productivity and Activity Impairment					
Absenteeism	466	2.32 ± 10.72 [0.00]	486	5.68 ± 16.28 [0.00]	< 0.001
Presenteeism	491	19.90 ± 24.95 [10.00]	501	25.09 ± 26.19 [20.00]	0.001
Total Work Productivity Impairment	464	20.79 ± 25.92 [10.00]	480	26.83 ± 27.92 [20.00]	< 0.001
Total Activity Impairment	1594	18.76 ± 23.85 [10.00]	1594	24.13 ± 26.09 [10.00]	< 0.001
Healthcare Resource Utilization					
No. of Physician Visits in the Past 6 Months	1594	5.51 ± 7.15 [3.00]	1594	7.44 ± 9.98 [5.00]	< 0.001
No. of ER Visits in the Past 6 Months	1594	0.09 ± 1.26 [0.00]	1594	0.22 ± 3.23 [0.00]	0.125
No. of Hospitalizations in the Past 6 Months	1594	0.37 ± 2.83 [0.00]	1594	0.67 ± 6.73 [0.00]	0.098
Cost					
Absenteeism Cost (thousand yen)	466	94.60 ± 474.40 [0.00]	486	206.23 ± 592.62 [0.00]	0.001
Presenteeism Cost (thousand yen)	491	759.65 ± 1010.13 [360.72]	501	938.64 ± 1034.25 [594.96]	0.006
Indirect Cost (thousand yen)	464	793.22 ± 1054.45 [360.72]	480	1002.63 ± 1098.34 [623.49]	0.003
Direct Cost (thousand yen)	1594	902.63 ± 6142.10 [52.01]	1594	1600.64 ± 14712.23 [86.69]	0.081

Categorical Variable	N	%	N	%	p-value^b^
PHQ-9					
PHQ-9 Score < 10	1507	94.5%	1465	91.9%	0.003
PHQ-9 Score ≥ 10	87	5.5%	129	8.1%	

a: p-value based on one-way ANOVA

b: p-value based on Pearson’s chi-square test

In the sensitivity analysis using 1:2 propensity matching, most of the key results were consistent with the 1:1 propensity matching results (Table 4). Caregivers of patients with Alzheimer’s disease/dementia had significantly more physician visits and a greater number of hospitalizations in the past 6 months, compared to non-caregivers (*p* < 0.01). However, the differences in the number of ER visits in the past 6 months were not statistically significant. In addition, caregivers of patients with Alzheimer’s disease/dementia had significantly higher absenteeism cost, presenteeism cost, indirect cost, and direct cost (*p* < 0.01) (data not shown).

Multivariable analyses adjusting for covariates were also conducted to evaluate the outcomes of these two groups. As shown in Table S6, after adjusting for potential confounding effects of demographics and clinical characteristic variables, caregivers of patients with Alzheimer’s disease/dementia scored significantly lower in MCS, PCS, EQ-5D, and SF-6D index compared to non-caregivers (*p* < 0.001). In addition, they had significantly greater absenteeism, presenteeism, total work productivity, and total activity impairment (*p* < 0.01), had more healthcare utilization and costs than non-caregivers (*p* < 0.05).

#### Stroke

The demographic data of the caregivers and non-caregivers after 1:1 propensity score matching is shown in Table S7.

All variables were balanced between caregivers of patients with stroke patients and non-caregivers after 1:1 propensity score matching.

After propensity score matching, outcome variables were compared between caregivers and their respective matched non-caregivers (Table 5). Caregivers of patients with stroke had significantly lower MCS, EQ-5D index, and SF-6D compared to non-caregivers (*p* < 0.05), however, the difference in PCS was not statistically significant. They had significantly greater impairment in total activity (*p* < 0.05) but not impairment in total work productivity compared to non-caregivers. They had significantly more ER visits in the past 6 months (*p* < 0.05), however, the differences in the number of physician visits and hospitalizations in the past 6 months were not statistically significant compared to non-caregivers. In addition, no significant differences were found in costs. There was no significant difference in PHQ-9 between caregivers of patients with stroke and non-caregivers.

**Table 5:** Outcome variables between caregiver of stroke and non-caregivers after propensity score matching 1:1

	Non-caregiver		Caregiver of Stroke Patients		
Continuous Variable	*N*	Mean ± SD [Median]	*N*	Mean ± SD [Median]	*p*-value^a^
Health-related Quality of Life					
Mental Component Summary (MCS)	342	48.00 ± 10.44 [49.32]	342	46.08 ± 10.79 [47.83]	0.018
Physical Component Summary (PCS)	342	51.97 ± 6.87 [53.39]	342	51.61 ± 6.67 [53.05]	0.488
EQ-5D	342	0.85 ± 0.17 [0.83]	342	0.82 ± 0.16 [0.81]	0.026
SF-6D	342	0.76 ± 0.13 [0.76]	342	0.73 ± 0.12 [0.72]	0.005
Work Productivity and Activity Impairment					
Absenteeism	107	2.37 ± 11.06 [0.00]	105	4.07 ± 13.80 [0.00]	0.325
Presenteeism	115	19.39 ± 25.00 [10.00]	109	24.68 ± 26.86 [10.00]	0.128
Total Work Productivity Impairment	106	20.73 ± 25.98 [10.00]	104	25.84 ± 28.44 [10.00]	0.175
Total Activity Impairment	342	21.35 ± 25.51 [10.00]	342	25.91 ± 28.38 [20.00]	0.027
Healthcare Resource Utilization					
No. of Physician Visits in the Past 6 Months	342	5.76 ± 10.28 [3.00]	342	6.33 ± 8.78 [4.00]	0.436
No. of ER Visits in the Past 6 Months	342	0.02 ± 0.13 [0.00]	342	0.15 ± 1.10 [0.00]	0.032
No. of Hospitalizations in the Past 6 Months	342	0.24 ± 2.14 [0.00]	342	0.67 ± 6.69 [0.00]	0.258
Cost					
Absenteeism Cost (thousand yen)	107	84.25 ± 364.60 [0.00]	105	155.29 ± 614.58 [0.00]	0.306
Presenteeism Cost (thousand yen)	115	750.02 ± 1023.66 [332.55]	109	925.50 ± 1066.83 [508.14]	0.210
Indirect Cost (thousand yen)	106	809.58 ± 1076.13 [361.39]	104	955.34 ± 1108.60 [509.67]	0.335
Direct Cost (thousand yen)	342	613.64 ± 4661.09 [52.01]	342	1562.23 ± 14480.98 [69.35]	0.249

Categorical Variable	N	%	N	%	p-value^b^
PHQ-9					
PHQ-9 Score < 10	313	91.5%	305	89.2%	0.300
PHQ-9 Score ≥ 10	29	8.5%	37	10.8%	

a: p-value based on one-way ANOVA

b: p-value based on Pearson’s chi-square test

In the sensitivity analysis using 1:2 propensity matching, most of the key results were consistent with the 1:1 propensity matching results (Table 5). Caregivers of patients with stroke were found to have higher presenteeism cost and indirect cost (*p* < 0.05) (data not shown).

Multivariable analyses adjusting for covariates were also conducted to evaluate the outcomes of these two groups. As shown in Table S8, after adjusting for potential confounding effects of demographics and clinical characteristic variables, caregivers of patients with stroke scored significantly lower in MCS and SF-6D index compared to the non-caregivers (*p* < 0.05). In addition, they had significantly greater total activity impairment and more direct cost (*p* < 0.05), however, there were no significant differences in healthcare utilization, indirect costs, and PHQ-9 compared to non-caregivers.

### Pairwise comparison of burden among caregivers of patients with schizophrenia, depression, Alzheimer’s disease/dementia, and stroke

**Fig. 1:**
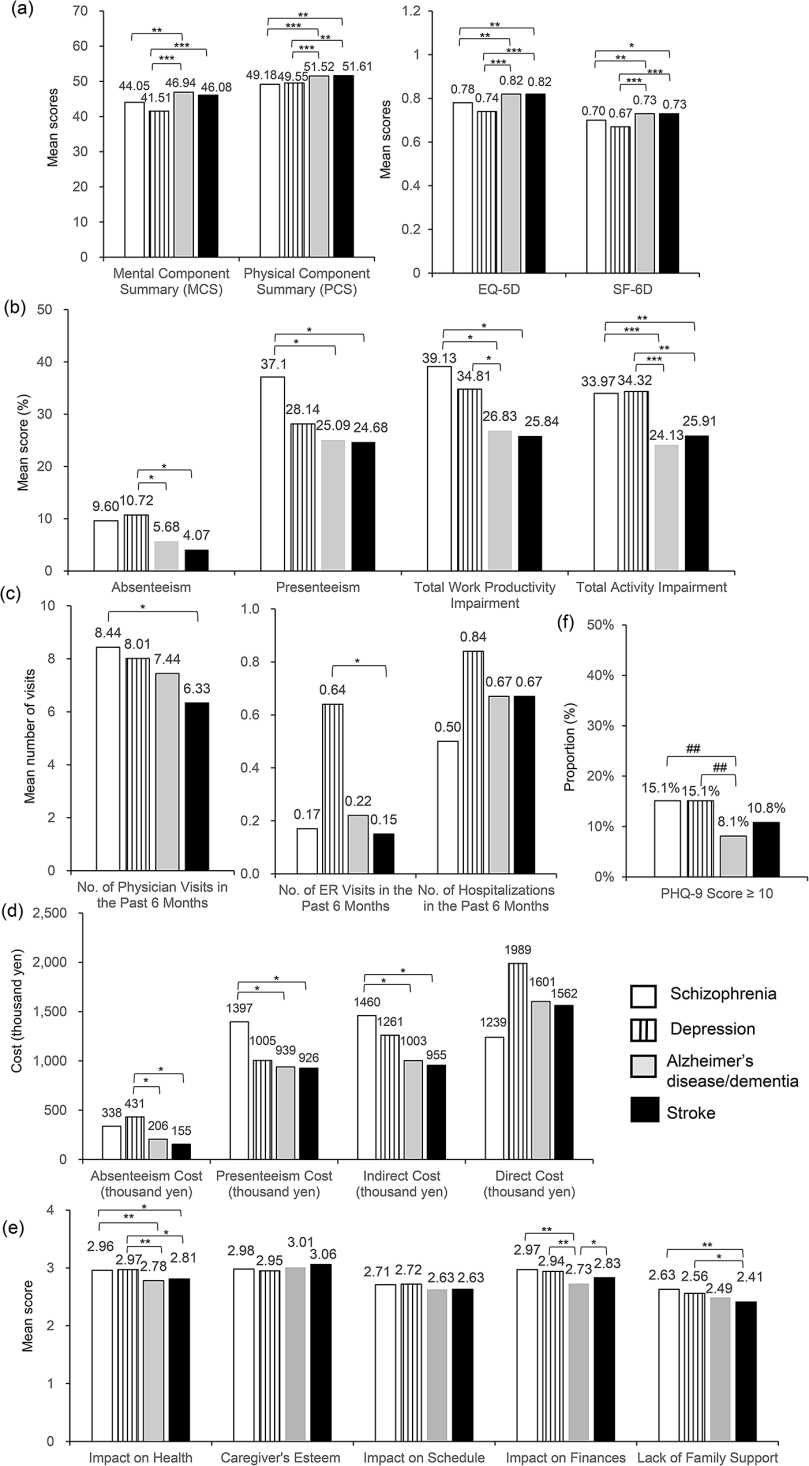
Pairwise comparison of outcome variables among the caregiver groups. **(a)** Health-related Quality of Life, **(b)** Work Productivity and Activity Impairment, **(c)** Healthcare Resource Utilization, **(d)** Cost, **(e)** Caregiver Reaction Assessment, **(f)** PHQ-9. *: *p* < 0.05, **p < 0.01, ***p < 0.001 (based on one-way ANOVA), ## *p* < 0.01 (Pearson’s chi-square test)

As shown in Fig. 1 and Table S9, outcomes were compared among the caregiver groups (pairwise comparison).

#### Caregivers of patients with Schizophrenia vs. caregivers of patients with Depression

Bivariate analyses revealed no significant differences in the burden between caregivers of patients with schizophrenia and caregivers of patients with depression in all aspects of HRQoL, WPAI, HRU, economic cost, CRA and PHQ-9.

#### Caregivers of patients with Schizophrenia vs. caregivers of patients with Alzheimer’s disease/dementia

Compared to caregivers of patients with Alzheimer’s disease/dementia, caregivers of patients with schizophrenia had significantly lower HRQoL scores (*p* < 0.01) and greater presenteeism, total work productivity impairment, and total activity impairment (*p* < 0.05).

In terms of economic costs, caregivers of patients with schizophrenia had significantly higher presenteeism cost and indirect cost compared to caregivers of patients with Alzheimer’s disease/dementia (*p* < 0.05). The differences in HRU and direct cost were not statistically significant compared to those of caregivers of patients with Alzheimer’s disease/dementia.

Caregivers of patients with schizophrenia had a significantly greater impact on health and greater impact on finances compared to caregivers of patients with Alzheimer’s disease/dementia (*p* < 0.01).

Caregivers of patients with schizophrenia also had a significantly higher proportion of PHQ-9 score ≥ 10 than caregivers of patients with Alzheimer’s disease/dementia (*p* < 0.01).

#### Caregivers of patients with Schizophrenia vs. caregivers of patients with Stroke

Caregivers of patients with schizophrenia had significantly lower HRQoL in terms of PCS, EQ-5D, and SF-6D (not MCS) than caregivers of patients with stroke (*p* < 0.05). Significantly greater presenteeism, total work productivity impairment, and total activity impairment were also reported in caregivers of patients with schizophrenia compared to caregivers of patients with stroke (*p* < 0.05). In terms of HRU, caregivers of patients with schizophrenia had more visits to physicians in the last 6 months than caregivers of patients with stroke (*p* < 0.05).

Additionally, the presenteeism cost and indirect cost were significantly higher among caregivers of patients with schizophrenia than caregivers of patients with stroke (*p* < 0.05).

Caregivers of patients with schizophrenia had higher impact on health (*p* < 0.05) and a higher degree of lack of family support in caregiving than caregivers of patients with stroke (*p* < 0.01).

No significant differences were identified in PHQ-9 between caregivers of patients with schizophrenia and caregivers of patients with stroke.

#### Caregivers of patients with Depression vs. caregivers of patients with Alzheimer’s disease/dementia

Caregivers of patients with depression had significantly lower PCS, MCS, EQ-5D index, and SF-6D compared to caregivers of patients with Alzheimer’s disease/dementia (*p* < 0.001). Caregivers of patients with depression had greater impairment in absenteeism, total work productivity, and total activity impairment than caregivers of patients with Alzheimer’s disease/dementia (*p* < 0.05), but the difference in presenteeism was not statistically significant.

Caregivers of patients with depression’s absenteeism cost were more than twice that of caregivers of patients with Alzheimer’s disease/dementia (*p* < 0.05), but the other costs and HRU were not significantly different.

Caregivers of patients with depression had a greater impact on health and impact on finances compared to caregivers of patients with Alzheimer’s disease/dementia (*p* < 0.01).

A significantly higher proportion of caregivers of patients with depression had PHQ-9 score ≥ 10 compared to caregivers of patients with Alzheimer’s disease/dementia (*p* < 0.01).

#### Caregivers of patients with Depression vs. caregivers of patients with Stroke

Caregivers of patients with depression were significantly lower in all HRQoL scales (MCS, PCS, EQ-5D, SF-6D) (*p* < 0.01) and greater in most work productivity-related items (absenteeism and total activity impairment) than caregivers of patients with stroke (*p* < 0.05).

Caregivers of patients with depression made more ER visits in the past 6 months and have higher absenteeism cost compared to caregivers of patients with stroke (*p* < 0.05).

Caregivers of patients with depression had a greater impact on health and a higher degree of lack of family support in caregiving than caregivers of patients with stroke (*p* < 0.05).

No significant difference in PHQ-9 was found between caregivers of patients with depression and caregivers of patients with stroke.

#### Caregivers of patients with Alzheimer’s disease/dementia vs. caregivers of patients with Stroke

There were no statistically significant differences in HRQoL, work productivity, activity impairment, HRU and costs between caregivers of patients with Alzheimer’s disease/dementia and caregivers of patients with stroke.

Compared to caregivers of patients with Alzheimer’s disease/dementia, caregivers of patients with stroke had a greater impact on finances (*p* < 0.05).

No significant difference in PHQ-9 was identified between caregivers of patients with Alzheimer’s disease/dementia and caregivers of patients with stroke.

## Discussion

Caregiving often causes chronic stress, which could negatively affect the physical and psychological health of caregivers [41]. Few studies have investigated and compared the impact of caring for patients with disorders that affect patients’ mental and/or cognitive functions on caregivers’ quality of life, burden, and economy in Japan. In this study, we provided insights into the burden of caregivers of patients with schizophrenia, depression, Alzheimer’s disease/dementia, and stroke in Japan and compared the caregiving burden across different conditions.

The average age of the caregivers of the four disorders assessed in this study ranged between 45.5 and 55.1 years. The caregivers of patients with depression were younger (45.5 years), while the caregivers of patients with dementia (55.1 years) were older than non-caregivers (51.7 years old). This finding is consistent with previous findings on caregivers in Japan [42]. There were slightly more female caregivers of patients with schizophrenia, depression, or stroke, possibly due to societal and cultural norms in Japan, where females were more often referred to for caregiving roles [43]. In contrast, the proportions of male and female caregivers were similar among caregivers of patients with Alzheimer’s disease/dementia [28, 42], which could be attributed to the higher prevalence of Alzheimer’s disease/dementia in females than in males [44].

Regardless of the nature of the disorder, caregivers experienced significantly lower HRQoL, greater HRU, higher economic burden (direct and indirect costs), greater impairment of total productivity (presenteeism and absenteeism) and total activity and a higher prevalence of depression (measured by PHQ-9 score ≥ 10). The study findings were highly reflective of the overall caregiver burden previously reported across different geographies irrespective of the different healthcare systems and cultures [14, 28, 45–50]. For instance, in the U.S., caregivers of patients with schizophrenia reported lower HRQoL and higher economic burden [49]. The few reports on the quality of life of caregivers of people with depression have demonstrated that caring for depressed elderly people could contribute to negative long-term health effects and an increased risk of death, and these individuals are more likely to suffer from depression. Studies evaluating the humanistic and economic burdens of caregivers of patients with Alzheimer’s disease/dementia have also reported similar findings, in which caregivers experienced poorer HRQoL, health state utility scores, total productivity and activity impairment [28, 42, 51]. Caregivers of patients with stroke patients residing in, but not limited to, Japan also reportedly have lower HRQoL [14, 46, 48]. Changes in Japan’s government care insurance policy have shifted care for disabled individuals (e.g., Alzheimer’s disease/dementia or stroke patients) relying on long-term care from hospital-based to community-based care [3], potentially resulting in increased family stress and future financial costs due to possible reduced formal care availability [52, 53].

Our study also compared the burden among caregivers of patients with schizophrenia, depression, Alzheimer’s disease/dementia, and stroke in Japan. Caregivers of patients with schizophrenia or depression had significantly poorer HRQoL, greater impairment of work productivity and activity, and higher prevalence of depression (PHQ-9 score ≥ 10) compared to caregivers of patients with Alzheimer’s disease/dementia or stroke. This finding contrasts with that of another study comparing caregivers of patients with schizophrenia, Alzheimer’s disease, and cancer, where caregivers of patients with Alzheimer’s disease patients experienced a similar burden to that of schizophrenia caregivers [54]. The caregiving burden of Alzheimer’s disease has been reported to be influenced by the severity of the disease [55, 56], which was not explored in this study and should be considered in future studies.

Consistent with the findings of previous studies [49, 57], caregivers of patients with schizophrenia had significantly greater impairment of work productivity and activity, despite the sample size of caregivers of patients with schizophrenia responding to the survey being smaller (*n* = 30–31) than that of caregivers of patients with other mental disorders (*n* = 54–480). Caregivers of patients with depression also had lower work productivity and higher absenteeism and presenteeism. Considering that the caregivers of depressed patients were younger (45.5 years old) and had the highest proportion of employment (67.8%), this might have an impact on work productivity and economic costs. Notably, the WPAI survey holds relevance for caregivers who are employed and lacks insight into individuals who are unemployed. In this study, we observed higher proportions of unemployed caregivers of patients with Alzheimer’s disease/dementia and stroke than caregivers of patients with schizophrenia and depression and may contribute to a greater overall societal economic burden. Future studies are warranted.

In our study, we used the CRA to investigate experiences with caregiving. Past studies using CRA reported relatively high responses to questions about caregiver self-esteem and low responses to questions indicating caregiver resentment in the different caregiver populations [58–62], which was also observed in different caregiver groups in our study. Caregivers of patients with schizophrenia and depression were more likely to perceive a loss in physical strength and a financial burden to the same extent as their self-esteem. These results suggested that informal caregivers want to care for patients and feel privileged in providing care, but caregiving affects caregivers both objectively and subjectively. Another previous study suggested that the severity in the symptoms of diseases also influenced the caregiver’s level of burden [63, 64], but this information on disease severity was not available in this study.

Both Alzheimer’s disease/dementia and stroke are associated with cognitive decline and may require complex caregiving [12, 13, 65]. It is possible that the availability and use of long-term care and welfare services in Japan could reduce the long-term care burden associated with Alzheimer’s disease/dementia and stroke [3, 66] compared to that associated with schizophrenia or depression. Furthermore, stigma against mental disorders, especially schizophrenia, is a major issue in Japan [67, 68], and stigma is associated with caregivers’ depression and quality of life [69]. Therefore, the high burden of caregivers of patients with schizophrenia and depression may be related to stigma.

Intriguingly, there were no significant differences in the burden between caregivers of patients with Alzheimer’s disease/dementia and caregivers of patients with stroke, which could be attributed to the level of disability and dependency caused by either condition [4, 70]. Previous studies have shown that the HRQoL of caregivers is a factor influencing patients’ symptoms [71, 72], wherein the decline of the caregiver’s mental health and quality of life could contribute to the risk of hospitalization or institutionalization of the patient. Therefore, it is important to improve the quality of life of caregivers and reduce the burden. This study provided insights into the caregiving burden of two psychiatric disorders and two neurological disorders, wherein, the burden of caring for patients with psychiatric disorders was greater than that of patients with Alzheimer’s disease/dementia and stroke. This indicates the need to provide support for caregivers of patients with psychiatric disorders to be better able to care for their loved ones. Not only improving the patient’s quality of life but also recovering caregiver’s quality of life and work productivity could need treatment to achieve remission and recovery.

## Limitations

Our study has some limitations because of a cross-sectional internet survey. First, our study is subjected to selection bias because participants in internet surveys were limited to those who had internet access. As such, they might not be representative of the wider population of caregivers of patients with schizophrenia, depression, Alzheimer’s disease/dementia, or stroke in Japan. Second, the diagnoses of patients with schizophrenia, depression, Alzheimer’s disease/dementia, or stroke in this study were self-reported by their caregivers. Third, no information on the severity of disease and treatment status of each patient and the relationship with each patient (e.g., parent, child, or spouse) was obtained in this survey. Fourth, this study excluded the samples who cared for the patients with multiple mental disorders or multiple patients to simplify the focus. In addition, the use of caregiving and welfare support services such as life helpers, home visit care nursing, daytime services, and facility services was unknown in this study. Finally, because the sample size of caregivers with schizophrenia and depression was small, it was not possible to adjust for background information when comparing caregivers.

## Conclusion

Our results showed that caregivers of patients with schizophrenia, depression, Alzheimer’s disease/dementia, and stroke experienced lower HRQoL, healthcare costs, and labor productivity compared to matched non-caregivers in Japan, which is consistent with the previous reports. Additionally, caregivers of patients with psychiatric disorders, such as schizophrenia and depression, were found to experience greater burden in terms of lower HRQoL and work productivity than caregivers of patients with neurological disorders (Alzheimer’s disease/dementia or stroke). Collectively, the findings indicated a need to provide support for caregivers of patients with psychiatric disorders as well as neurological disorders in Japan to be better able to care for their patients.

### Electronic supplementary material

Below is the link to the electronic supplementary material.

Supplementary Material 1

## Data Availability

Study data to support our findings are available from Oracle Life Sciences (formerly known as Cerner Enviza), but availability of the data is restricted and was used under license for this study and are not publicly available. Data are however available from the authors upon reasonable request and with permission of Oracle Life Sciences (formerly known as Cerner Enviza).

## Abbreviations

ANOVA: Analysis of variance
BMI: Body mass index
CCI: Charlson Comorbidity Index
CRA: Caregiver reaction assessment
EQ-5D-5L: Five-Level EuroQol Five-Dimension
ER: Emergency room
GLMs: Generalized linear models
HRQoL: Health-related quality of life
HRU: Healthcare resource utilization
IQR: Interquartile range
LSR: Lightspeed Research
MCS: Mental component summary
NHWS: National Health and Wellness Survey
PCS: Physical component summary
PHQ-9: Patient Health Questionnaire-9
SF-6D: Short-Form Six-Dimension
SF-12v2: 12-item Short-Form Health Survey version 2
SMD: Standardized mean difference
US: United States
WPAI: Work productivity and activity impairment
YLDs: Years lived with disability
