# Supplementary material for: Burden in caregivers of patients with schizophrenia, depression, dementia, and stroke in Japan: comparative analysis of quality of life, work productivity, and qualitative caregiving burden
Source: BMC Psychiatry. 2024 Sep 2;24:591. doi: 10.1186/s12888-024-06000-x (PMC11370303; doi:10.1186/s12888-024-06000-x)
Supplement: Supplementary file 1 — Supplementary Material 1 [file 12888_2024_6000_MOESM1_ESM.docx]

**Table S1 Demographic and general health characteristics between caregivers of schizophrenia and non-caregivers (post-match)**

|  | | **Non-caregiver** | | **Caregiver of Schizophrenia patients** | |  |  |
| --- | --- | --- | --- | --- | --- | --- | --- |
| **Continuous Variable** | | **N** | **Mean, SD, Median, IQR, Range** | **N** | **Mean, SD, Median, IQR, Range** | **p-value** | **SMD** |
| Age | | 126 | 50.32, 16.94, 52.00, 31.00, 61.00 | 126 | 51.75, 16.83, 53.00, 30.00, 63.00 | 0.503 | 0.085 |
| Charlson Comorbidity Index | | 126 | 0.37, 1.06, 0.00, 0.00, 9.00 | 126 | 0.39, 1.40, 0.00, 0.00, 14.00 | 0.879 | 0.019 |
| **Categorical Variable** | | **N** | **%** | **N** | **%** | **p-value** | **SMD** |
| Gender | *Male* | 62 | 49.2% | 60 | 47.6% | 0.801 | 0.032 |
|  | *Female* | 64 | 50.8% | 66 | 52.4% |  |  |
| Marital Status | *Married or living with partner* | 74 | 58.7% | 71 | 56.3% | 0.702 | 0.048 |
|  | *Not Married* | 52 | 41.3% | 55 | 43.7% |  |  |
|  | *Decline to answer* | 0 | 0.0% | 0 | 0.0% |  |  |
| Level of Education | *Completed university education* | 36 | 28.6% | 41 | 32.5% | 0.465 | 0.156 |
|  | *Not* | 90 | 71.4% | 84 | 66.7% |  |  |
|  | *Decline to answer* | 1 | 0.0% | 1 | 0.8% |  |  |
| Household Income | *< ¥3,000,000* | 32 | 25.4% | 32 | 25.4% | 0.906 | 0.128 |
|  | *¥3,000,000 to < ¥5,000,000* | 38 | 30.2% | 37 | 29.4% |  |  |
|  | *¥5,000,000 to < ¥8,000,000* | 27 | 21.4% | 29 | 23.0% |  |  |
|  | *¥8,000,000 or more* | 15 | 11.9% | 18 | 14.3% |  |  |
|  | *Decline to answer* | 14 | 11.1% | 10 | 7.9% |  |  |
| Employment Status | *Currently employed* | 71 | 56.3% | 72 | 57.1% | 0.899 | 0.016 |
|  | *Not* | 55 | 43.7% | 54 | 42.9% |  |  |
| Body Mass Index | *Underweight (BMI < 18.5)* | 27 | 21.4% | 23 | 18.3% | 0.896 | 0.098 |
|  | *Normal (BMI >=18.5 & <25)* | 66 | 52.4% | 68 | 54.0% |  |  |
|  | *Obese (BMI >=25)* | 26 | 20.6% | 26 | 20.6% |  |  |
|  | *Decline to answer* | 7 | 5.6% | 9 | 7.1% |  |  |
| Smoking Status | *Never* | 62 | 49.2% | 62 | 49.2% | 0.984 | 0.023 |
|  | *Former* | 40 | 31.7% | 39 | 31.0% |  |  |
|  | *Current* | 24 | 19.0% | 25 | 19.8% |  |  |
| Alcohol Use | *≤ once per week* | 81 | 64.3% | 82 | 65.1% | 0.895 | 0.017 |
|  | *≥ 2-3 times per week* | 45 | 35.7% | 44 | 34.9% |  |  |
| Vigorous Exercise in Past 30 Days | *0-11 times* | 102 | 81.0% | 100 | 79.4% | 0.752 | 0.040 |
|  | *≥ 12 times* | 24 | 19.0% | 26 | 20.6% |  |  |
| p-value: Pearson’s Chi-square test was used for categorical variables and one-way ANOVA for continuous variables. | | | | | | |  |

**Table S2 Adjusted means with 95% CIs of the outcomes for caregivers of schizophrenia and non-caregivers**

|  | **Non-caregiver (N=126)** | | | | **Caregiver of Schizophrenia (N=126)** | | | | **Difference** | | | | **p-value** |  |
| --- | --- | --- | --- | --- | --- | --- | --- | --- | --- | --- | --- | --- | --- | --- |
|  | **(1)** | | | | **(2)** | | | | **(2)-(1)** | | | |  |  |
|  | **Mean** | **SE** | **95% LCL** | **95% UCL** | **Mean** | **SE** | **95% LCL** | **95% UCL** | **Mean** | **SE** | **95% LCL** | **95% UCL** | **(1) vs. (2)** |  |
|  |  |  |  |  |  |  |  |  |  |  |  |  |  |  |
| PHQ-9 ≥ 10 | 0.11 | 0.05 | 0.05 | 0.24 | 0.10 | 0.04 | 0.04 | 0.23 | -0.01 | 0.04 | -0.08 | 0.07 | 0.887 |  |
| Mental component summary (MCS) | 47.35 | 1.21 | 44.98 | 49.72 | 44.29 | 1.17 | 42.00 | 46.58 | -3.06 | 1.25 | -5.52 | -0.61 | 0.015 |  |
| Physical component summary (PCS) | 53.10 | 0.86 | 51.42 | 54.79 | 50.25 | 0.83 | 48.63 | 51.88 | -2.85 | 0.89 | -4.59 | -1.11 | 0.001 |  |
| EQ-5D | 0.86 | 0.02 | 0.82 | 0.90 | 0.80 | 0.02 | 0.76 | 0.83 | -0.06 | 0.02 | -0.10 | -0.02 | 0.002 |  |
| SF-6D | 0.75 | 0.02 | 0.72 | 0.78 | 0.71 | 0.01 | 0.68 | 0.74 | -0.05 | 0.02 | -0.08 | -0.01 | 0.004 |  |
| Absenteeism | 3.94 | 2.30 | 1.25 | 12.38 | 6.69 | 4.16 | 1.98 | 22.60 | 2.75 | 4.84 | -6.73 | 12.23 | 0.543 |  |
| Presenteeism | 23.29 | 4.33 | 16.18 | 33.54 | 36.29 | 7.46 | 24.26 | 54.29 | 13.00 | 9.01 | -4.65 | 30.65 | 0.128 |  |
| Total work productivity impairment | 21.04 | 4.41 | 13.95 | 31.72 | 38.04 | 8.50 | 24.55 | 58.95 | 17.01 | 9.89 | -2.38 | 36.39 | 0.063 |  |
| Total activity Impairment | 20.39 | 2.71 | 15.71 | 26.46 | 29.10 | 3.68 | 22.71 | 37.29 | 8.71 | 3.55 | 1.76 | 15.67 | 0.011 |  |
| No. of Physician visits in the Past 6 Months | 4.56 | 0.71 | 3.36 | 6.19 | 8.46 | 1.29 | 6.27 | 11.40 | 3.90 | 1.19 | 1.58 | 6.22 | < 0.001 |  |
| Visited ER in the Past 6 Months | 0.01 | 0.01 | 0.00 | 0.04 | 0.04 | 0.02 | 0.01 | 0.12 | 0.03 | 0.02 | -0.01 | 0.07 | 0.023 |  |
| Hospitalized in the Past 6 Months | 0.02 | 0.02 | 0.00 | 0.10 | 0.12 | 0.06 | 0.05 | 0.28 | 0.10 | 0.05 | 0.00 | 0.20 | 0.015 |  |
| Absenteeism cost (in thousand ¥) | 106.86 | 63.36 | 33.43 | 341.58 | 245.66 | 155.41 | 71.10 | 848.83 | 138.80 | 169.67 | -193.75 | 471.35 | 0.344 |  |
| Presenteeism cost (in thousand ¥) | 807.76 | 148.37 | 563.55 | 1157.80 | 1296.49 | 264.00 | 869.85 | 1932.39 | 488.73 | 313.95 | -126.61 | 1104.06 | 0.098 |  |
| Indirect cost (in thousand ¥) | 705.59 | 147.49 | 468.42 | 1062.87 | 1362.95 | 305.14 | 878.84 | 2113.73 | 657.36 | 348.07 | -24.84 | 1339.56 | 0.038 |  |
| Direct cost (in thousand ¥) | 300.32 | 143.74 | 117.54 | 767.35 | 1268.08 | 575.80 | 520.77 | 3087.82 | 967.76 | 530.72 | -72.43 | 2007.95 | 0.004 |  |
| SE: Standard Error, LCL: Lower Confidence Limit, UCL: Upper Confidence Limit | | | | |  |  |  |  |  |  |  |  |  |  |

**Table S3 Demographic and general health characteristics between caregivers of depression and non-caregivers (post-match)**

|  | | **Non-caregiver** | | **Caregiver of Depression patients** | |  |  |
| --- | --- | --- | --- | --- | --- | --- | --- |
| **Continuous Variable** | | **N** | **Mean, SD, Median, IQR, Range** | **N** | **Mean, SD, Median, IQR, Range** | **p-value** | **SMD** |
| Age | | 146 | 45.75, 16.96, 46.00, 31.00, 62.00 | 146 | 45.45, 17.01, 43.00, 29.00, 59.00 | 0.880 | 0.018 |
| Charlson Comorbidity Index | | 146 | 0.26, 0.66, 0.00, 0.00, 4.00 | 146 | 0.31, 0.72, 0.00, 0.00, 4.00 | 0.555 | 0.069 |
| **Categorical Variable** | | **N** | **%** | **N** | **%** | **p-value** | **SMD** |
| Gender | *Male* | 62 | 42.5% | 68 | 46.6% | 0.480 | 0.083 |
|  | *Female* | 84 | 57.5% | 78 | 53.4% |  |  |
| Marital Status | *Married or living with partner* | 86 | 58.9% | 84 | 57.5% | 0.361 | 0.168 |
|  | *Not Married* | 56 | 38.4% | 61 | 41.8% |  |  |
|  | *Decline to answer* | 4 | 2.7% | 1 | 0.7% |  |  |
| Level of Education | *Completed university education* | 63 | 43.2% | 68 | 46.6% | 0.764 | 0.086 |
|  | *Not* | 79 | 54.1% | 73 | 50.0% |  |  |
|  | *Decline to answer* | 4 | 2.7% | 5 | 3.4% |  |  |
| Household Income | *< ¥3,000,000* | 37 | 25.3% | 34 | 23.3% | 0.833 | 0.142 |
|  | *¥3,000,000 to < ¥5,000,000* | 31 | 21.2% | 35 | 24.0% |  |  |
|  | *¥5,000,000 to < ¥8,000,000* | 40 | 27.4% | 38 | 26.0% |  |  |
|  | *¥8,000,000 or more* | 31 | 21.2% | 28 | 19.2% |  |  |
|  | *Decline to answer* | 7 | 4.8% | 11 | 7.5% |  |  |
| Employment Status | *Currently employed* | 93 | 63.7% | 99 | 67.8% | 0.459 | 0.087 |
|  | *Not* | 53 | 36.3% | 47 | 32.2% |  |  |
| Body Mass Index | *Underweight (BMI < 18.5)* | 9 | 6.2% | 12 | 8.2% | 0.896 | 0.091 |
|  | *Normal (BMI >=18.5 & <25)* | 100 | 68.5% | 97 | 66.4% |  |  |
|  | *Obese (BMI >=25)* | 33 | 22.6% | 32 | 21.9% |  |  |
|  | *Decline to answer* | 4 | 2.7% | 5 | 3.4% |  |  |
| Smoking Status | *Never* | 82 | 56.2% | 77 | 52.7% | 0.810 | 0.076 |
|  | *Former* | 30 | 20.5% | 34 | 23.3% |  |  |
|  | *Current* | 34 | 23.3% | 35 | 24.0% |  |  |
| Alcohol Use | *≤ once per week* | 97 | 66.4% | 88 | 60.3% | 0.274 | 0.128 |
|  | *≥ 2-3 times per week* | 49 | 33.6% | 58 | 39.7% |  |  |
| Vigorous Exercise in Past 30 Days | *0-11 times* | 116 | 79.5% | 113 | 77.4% | 0.670 | 0.050 |
|  | *≥ 12 times* | 30 | 20.5% | 33 | 22.6% |  |  |
| p-value: Pearson’s Chi-square test was used for categorical variables and one-way ANOVA for continuous variables. | | | | | | |  |

**Table S4 Adjusted means with 95% CIs of the outcomes for caregivers of depression and non-caregivers**

|  | **Non-caregiver (N=146)** | | | | **Caregiver of Depression (N=146)** | | | | **Difference** | | | | **p-value** |  |
| --- | --- | --- | --- | --- | --- | --- | --- | --- | --- | --- | --- | --- | --- | --- |
|  | **(1)** | | | | **(2)** | | | | **(2)-(1)** | | | |  |  |
|  | **Mean** | **SE** | **95% LCL** | **95% UCL** | **Mean** | **SE** | **95% LCL** | **95% UCL** | **Mean** | **SE** | **95% LCL** | **95% UCL** | **(1) vs. (2)** |  |
|  |  |  |  |  |  |  |  |  |  |  |  |  |  |  |
| PHQ-9 ≥ 10 | 0.08 | 0.03 | 0.03 | 0.16 | 0.14 | 0.04 | 0.07 | 0.25 | 0.06 | 0.04 | -0.01 | 0.13 | 0.075 |  |
| Mental component summary (MCS) | 47.45 | 1.12 | 45.25 | 49.66 | 40.89 | 1.09 | 38.76 | 43.02 | -6.56 | 1.12 | -8.76 | -4.37 | < 0.001 |  |
| Physical component summary (PCS) | 51.18 | 0.77 | 49.68 | 52.68 | 48.91 | 0.74 | 47.46 | 50.36 | -2.27 | 0.76 | -3.76 | -0.78 | 0.003 |  |
| EQ-5D | 0.85 | 0.02 | 0.81 | 0.88 | 0.73 | 0.02 | 0.69 | 0.76 | -0.12 | 0.02 | -0.15 | -0.08 | < 0.001 |  |
| SF-6D | 0.75 | 0.01 | 0.72 | 0.77 | 0.66 | 0.01 | 0.64 | 0.69 | -0.09 | 0.01 | -0.11 | -0.06 | < 0.001 |  |
| Absenteeism | 3.30 | 1.40 | 1.44 | 7.56 | 7.90 | 3.49 | 3.32 | 18.80 | 4.60 | 3.87 | -2.97 | 12.18 | 0.170 |  |
| Presenteeism | 13.19 | 2.19 | 9.52 | 18.27 | 28.59 | 4.72 | 20.68 | 39.52 | 15.40 | 5.22 | 5.17 | 25.63 | < 0.001 |  |
| Total work productivity impairment | 15.76 | 2.71 | 11.24 | 22.09 | 35.45 | 6.21 | 25.15 | 49.96 | 19.69 | 6.78 | 6.40 | 32.97 | < 0.001 |  |
| Total activity Impairment | 19.52 | 2.79 | 14.75 | 25.83 | 37.02 | 5.00 | 28.41 | 48.24 | 17.50 | 4.41 | 8.86 | 26.15 | < 0.001 |  |
| No. of Physician visits in the Past 6 months | 5.56 | 0.80 | 4.20 | 7.37 | 9.74 | 1.31 | 7.49 | 12.67 | 4.18 | 1.17 | 1.89 | 6.48 | < 0.001 |  |
| Visited ER in the Past 6 Months | 0.05 | 0.03 | 0.02 | 0.13 | 0.12 | 0.05 | 0.05 | 0.24 | 0.07 | 0.04 | -0.01 | 0.15 | 0.062 |  |
| Hospitalized in the Past 6 Months | 0.03 | 0.02 | 0.01 | 0.08 | 0.09 | 0.03 | 0.04 | 0.17 | 0.06 | 0.03 | 0.01 | 0.11 | 0.018 |  |
| Absenteeism cost (in thousand ¥) | 118.71 | 50.25 | 51.78 | 272.16 | 272.05 | 119.34 | 115.15 | 642.74 | 153.34 | 132.56 | -106.46 | 413.15 | 0.188 |  |
| Presenteeism cost (in thousand ¥) | 489.81 | 88.98 | 343.08 | 699.29 | 993.04 | 179.09 | 697.36 | 1414.10 | 503.23 | 200.11 | 111.03 | 895.44 | 0.006 |  |
| Indirect cost (in thousand ¥) | 582.23 | 109.10 | 403.27 | 840.61 | 1231.84 | 233.84 | 849.13 | 1787.05 | 649.61 | 257.76 | 144.40 | 1154.81 | 0.005 |  |
| Direct cost (in thousand ¥) | 465.59 | 157.44 | 239.97 | 903.33 | 1297.09 | 438.30 | 668.87 | 2515.35 | 831.49 | 409.32 | 29.24 | 1633.74 | 0.007 |  |
| SE: Standard Error, LCL: Lower Confidence Limit, UCL: Upper Confidence Limit | | | | |  |  |  |  |  |  |  |  |  |  |

**Table S5 Demographic and general health characteristics between caregivers of Alzheimer's disease/dementia and non-caregivers (post-match)**

|  | | **Non-caregiver** | | **Caregiver of Alzheimer's disease/dementia** | |  |  |
| --- | --- | --- | --- | --- | --- | --- | --- |
| **Continuous Variable** | | **N** | **Mean, SD, Median, IQR, Range** | **N** | **Mean, SD, Median, IQR, Range** | **p-value** | **SMD** |
| Age | | 1594 | 56.31, 15.25, 60.00, 21.00, 84.00 | 1594 | 55.05, 15.02, 59.00, 21.00, 70.00 | 0.019 | 0.083 |
| Charlson Comorbidity Index | | 1594 | 0.26, 0.66, 0.00, 0.00, 8.00 | 1594 | 0.29, 0.69, 0.00, 0.00, 10.00 | 0.199 | 0.045 |
| **Categorical Variable** | | **N** | **%** | **N** | **%** | **p-value** | **SMD** |
| Gender | *Male* | 814 | 51.1% | 799 | 50.1% | 0.595 | 0.019 |
|  | *Female* | 780 | 48.9% | 795 | 49.9% |  |  |
| Marital Status | *Married or living with partner* | 1146 | 71.9% | 1109 | 69.6% | 0.150 | 0.051 |
|  | *Not Married* | 448 | 28.1% | 485 | 30.4% |  |  |
|  | *Decline to answer* | 0 | 0.0% | 0 | 0.0% |  |  |
| Level of Education | *Completed university education* | 767 | 48.1% | 785 | 49.2% | 0.720 | 0.029 |
|  | *Not* | 818 | 51.3% | 798 | 50.1% |  |  |
|  | *Decline to answer* | 9 | 0.6% | 11 | 0.7% |  |  |
| Household Income | *< ¥3,000,000* | 217 | 13.6% | 237 | 14.9% | 0.817 | 0.044 |
|  | *¥3,000,000 to < ¥5,000,000* | 392 | 24.6% | 386 | 24.2% |  |  |
|  | *¥5,000,000 to < ¥8,000,000* | 406 | 25.5% | 394 | 24.7% |  |  |
|  | *¥8,000,000 or more* | 399 | 25.0% | 408 | 25.6% |  |  |
|  | *Decline to answer* | 180 | 11.3% | 169 | 10.6% |  |  |
| Employment Status | *Currently employed* | 868 | 54.5% | 893 | 56.0% | 0.373 | 0.032 |
|  | *Not* | 726 | 45.5% | 701 | 44.0% |  |  |
| Body Mass Index | *Underweight (BMI < 18.5)* | 105 | 6.6% | 146 | 9.2% | 0.011 | 0.118 |
|  | *Normal (BMI >=18.5 & <25)* | 1150 | 72.1% | 1076 | 67.5% |  |  |
|  | *Obese (BMI >=25)* | 308 | 19.3% | 333 | 20.9% |  |  |
|  | *Decline to answer* | 31 | 1.9% | 39 | 2.4% |  |  |
| Smoking Status | *Never* | 852 | 53.5% | 836 | 52.4% | 0.273 | 0.057 |
|  | *Former* | 460 | 28.9% | 441 | 27.7% |  |  |
|  | *Current* | 282 | 17.7% | 317 | 19.9% |  |  |
| Alcohol Use | *≤ once per week* | 894 | 56.1% | 913 | 57.3% | 0.497 | 0.024 |
|  | *≥ 2-3 times per week* | 700 | 43.9% | 681 | 42.7% |  |  |
| Vigorous Exercise in Past 30 Days | *0-11 times* | 1200 | 75.3% | 1204 | 75.5% | 0.869 | 0.006 |
|  | *≥ 12 times* | 394 | 24.7% | 390 | 24.5% |  |  |
| p-value: Pearson’s Chi-square test was used for categorical variables and one-way ANOVA for continuous variables. | | | | | | |  |

**Table S6 Adjusted means with 95% CIs of the outcomes for caregivers of Alzheimer's disease/dementia and non-caregivers**

|  | **Non-caregiver (N=1594)** | | | | **Caregiver of Alzheimer's disease/ dementia (N=1594)** | | | | **Difference** | | | | **p-value** |  |
| --- | --- | --- | --- | --- | --- | --- | --- | --- | --- | --- | --- | --- | --- | --- |
|  | **(1)** | | | | **(2)** | | | | **(2)-(1)** | | | |  |  |
|  | **Mean** | **SE** | **95% LCL** | **95% UCL** | **Mean** | **SE** | **95% LCL** | **95% UCL** | **Mean** | **SE** | **95% LCL** | **95% UCL** | **(1) vs. (2)** |  |
|  |  |  |  |  |  |  |  |  |  |  |  |  |  |  |
| PHQ-9 ≥ 10 | 0.05 | 0.01 | 0.04 | 0.06 | 0.07 | 0.01 | 0.05 | 0.08 | 0.02 | 0.01 | 0.00 | 0.03 | 0.022 |  |
| Mental component summary (MCS) | 48.85 | 0.32 | 48.22 | 49.48 | 46.80 | 0.31 | 46.20 | 47.41 | -2.05 | 0.32 | -2.68 | -1.41 | < 0.001 |  |
| Physical component summary (PCS) | 52.28 | 0.20 | 51.88 | 52.67 | 51.55 | 0.20 | 51.16 | 51.93 | -0.73 | 0.21 | -1.13 | -0.33 | < 0.001 |  |
| EQ-5D | 0.86 | 0.01 | 0.85 | 0.87 | 0.82 | 0.01 | 0.81 | 0.83 | -0.04 | 0.01 | -0.05 | -0.03 | < 0.001 |  |
| SF-6D | 0.77 | 0.00 | 0.76 | 0.78 | 0.73 | 0.00 | 0.72 | 0.74 | -0.04 | 0.00 | -0.04 | -0.03 | < 0.001 |  |
| Absenteeism | 2.24 | 0.42 | 1.56 | 3.23 | 4.89 | 0.88 | 3.44 | 6.97 | 2.65 | 0.96 | 0.76 | 4.55 | 0.002 |  |
| Presenteeism | 20.26 | 1.09 | 18.23 | 22.52 | 24.69 | 1.32 | 22.24 | 27.41 | 4.42 | 1.67 | 1.14 | 7.71 | 0.008 |  |
| Total work productivity impairment | 21.13 | 1.17 | 18.97 | 23.55 | 26.21 | 1.42 | 23.56 | 29.15 | 5.07 | 1.81 | 1.54 | 8.61 | 0.005 |  |
| Total activity Impairment | 19.03 | 0.79 | 17.53 | 20.65 | 23.74 | 0.97 | 21.92 | 25.71 | 4.71 | 0.93 | 2.89 | 6.54 | < 0.001 |  |
| No. of Physician visits in the Past 6 months | 5.40 | 0.25 | 4.94 | 5.90 | 7.52 | 0.33 | 6.90 | 8.19 | 2.12 | 0.31 | 1.52 | 2.72 | < 0.001 |  |
| Visited ER in the Past 6 Months | 0.02 | 0.00 | 0.02 | 0.04 | 0.05 | 0.01 | 0.03 | 0.06 | 0.02 | 0.01 | 0.01 | 0.04 | < 0.001 |  |
| Hospitalized in the Past 6 Months | 0.05 | 0.01 | 0.04 | 0.07 | 0.09 | 0.01 | 0.07 | 0.11 | 0.04 | 0.01 | 0.02 | 0.06 | < 0.001 |  |
| Absenteeism cost (in thousand ¥) | 85.48 | 16.65 | 58.36 | 125.21 | 172.88 | 32.47 | 119.64 | 249.80 | 87.39 | 36.04 | 16.75 | 158.04 | 0.008 |  |
| Presenteeism cost (in thousand ¥) | 763.45 | 43.14 | 683.41 | 852.85 | 911.44 | 51.10 | 816.60 | 1017.31 | 148.00 | 65.41 | 19.79 | 276.21 | 0.023 |  |
| Indirect cost (in thousand ¥) | 795.97 | 46.03 | 710.68 | 891.49 | 964.65 | 55.05 | 862.56 | 1078.82 | 168.68 | 70.32 | 30.86 | 306.50 | 0.016 |  |
| Direct cost (in thousand ¥) | 792.09 | 175.12 | 513.55 | 1221.70 | 1364.17 | 274.31 | 919.83 | 2023.15 | 572.08 | 253.42 | 75.39 | 1068.77 | 0.016 |  |
| SE: Standard Error, LCL: Lower Confidence Limit, UCL: Upper Confidence Limit | | | | |  |  |  |  |  |  |  |  |  |  |

**Table S7 Demographic and general health characteristics between caregivers of stroke and non-caregivers (post-match)**

|  | | **Non-caregiver** | | **Caregiver of Stroke patients** | |  |  |
| --- | --- | --- | --- | --- | --- | --- | --- |
| **Continuous Variable** | | **N** | **Mean, SD, Median, IQR, Range** | **N** | **Mean, SD, Median, IQR, Range** | **p-value** | **SMD** |
| Age | | 342 | 53.38, 15.42, 54.00, 25.00, 69.00 | 342 | 53.19, 15.82, 55.00, 25.00, 65.00 | 0.877 | 0.044 |
| Charlson Comorbidity Index | | 342 | 0.24, 0.67, 0.00, 0.00, 7.00 | 342 | 0.28, 0.67, 0.00, 0.00, 7.00 | 0.496 | 0.035 |
| **Categorical Variable** | | **N** | **%** | **N** | **%** | **p-value** | **SMD** |
| Gender | *Male* | 151 | 44.2% | 154 | 45.0% | 0.817 | 0.070 |
|  | *Female* | 191 | 55.8% | 188 | 55.0% |  |  |
| Marital Status | *Married or living with partner* | 232 | 67.8% | 218 | 63.7% | 0.259 | 0.099 |
|  | *Not Married* | 110 | 32.2% | 124 | 36.3% |  |  |
|  | *Decline to answer* | 0 | 0.0% | 0 | 0.0% |  |  |
| Level of Education | *Completed university education* | 139 | 40.6% | 136 | 39.8% | 0.973 | 0.060 |
|  | *Not* | 202 | 59.1% | 205 | 59.9% |  |  |
|  | *Decline to answer* | 1 | 0.3% | 1 | 0.3% |  |  |
| Household Income | *< ¥3,000,000* | 71 | 20.8% | 79 | 23.1% | 0.968 | 0.099 |
|  | *¥3,000,000 to < ¥5,000,000* | 98 | 28.7% | 95 | 27.8% |  |  |
|  | *¥5,000,000 to < ¥8,000,000* | 61 | 17.8% | 60 | 17.5% |  |  |
|  | *¥8,000,000 or more* | 77 | 22.5% | 74 | 21.6% |  |  |
|  | *Decline to answer* | 35 | 10.2% | 34 | 9.9% |  |  |
| Employment Status | *Currently employed* | 201 | 58.8% | 192 | 56.1% | 0.486 | 0.030 |
|  | *Not* | 141 | 41.2% | 150 | 43.9% |  |  |
| Body Mass Index | *Underweight (BMI < 18.5)* | 30 | 8.8% | 31 | 9.1% | 0.824 | 0.080 |
|  | *Normal (BMI >=18.5 & <25)* | 220 | 64.3% | 223 | 65.2% |  |  |
|  | *Obese (BMI >=25)* | 75 | 21.9% | 76 | 22.2% |  |  |
|  | *Decline to answer* | 17 | 5.0% | 12 | 3.5% |  |  |
| Smoking Status | *Never* | 210 | 61.4% | 196 | 57.3% | 0.505 | 0.018 |
|  | *Former* | 69 | 20.2% | 80 | 23.4% |  |  |
|  | *Current* | 63 | 18.4% | 66 | 19.3% |  |  |
| Alcohol Use | *≤ once per week* | 226 | 66.1% | 220 | 64.3% | 0.630 | 0.049 |
|  | *≥ 2-3 times per week* | 116 | 33.9% | 122 | 35.7% |  |  |
| Vigorous Exercise in Past 30 Days | *0-11 times* | 295 | 86.3% | 291 | 85.1% | 0.662 | 0.025 |
|  | *≥ 12 times* | 47 | 13.7% | 51 | 14.9% |  |  |
| p-value: Pearson’s Chi-square test was used for categorical variables and one-way ANOVA for continuous variables. | | | | | | |  |

**Table S8 Adjusted means with 95% CIs of the outcomes for caregivers of stroke and non-caregivers**

|  | **Non-caregiver (N=342)** | | | | **Caregiver of Stroke (N=342)** | | | | **Difference** | | | | **p-value** |  |
| --- | --- | --- | --- | --- | --- | --- | --- | --- | --- | --- | --- | --- | --- | --- |
|  | **(1)** | | | | **(2)** | | | | **(2)-(1)** | | | |  |  |
|  | **Mean** | **SE** | **95% LCL** | **95% UCL** | **Mean** | **SE** | **95% LCL** | **95% UCL** | **Mean** | **SE** | **95% LCL** | **95% UCL** | **(1) vs. (2)** |  |
|  |  |  |  |  |  |  |  |  |  |  |  |  |  |  |
| PHQ-9 ≥ 10 | 0.04 | 0.02 | 0.02 | 0.10 | 0.06 | 0.02 | 0.03 | 0.12 | 0.01 | 0.01 | -0.02 | 0.04 | 0.456 |  |
| Mental component summary (MCS) | 47.30 | 0.78 | 45.78 | 48.82 | 45.60 | 0.76 | 44.12 | 47.08 | -1.70 | 0.75 | -3.16 | -0.23 | 0.023 |  |
| Physical component summary (PCS) | 52.20 | 0.51 | 51.19 | 53.20 | 51.94 | 0.50 | 50.96 | 52.92 | -0.25 | 0.49 | -1.22 | 0.72 | 0.610 |  |
| EQ-5D | 0.85 | 0.01 | 0.82 | 0.87 | 0.82 | 0.01 | 0.80 | 0.85 | -0.02 | 0.01 | -0.05 | 0.00 | 0.052 |  |
| SF-6D | 0.75 | 0.01 | 0.73 | 0.77 | 0.73 | 0.01 | 0.71 | 0.75 | -0.02 | 0.01 | -0.04 | -0.01 | 0.009 |  |
| Absenteeism | 2.35 | 0.89 | 1.12 | 4.94 | 3.25 | 1.27 | 1.51 | 7.00 | 0.90 | 1.60 | -2.24 | 4.03 | 0.566 |  |
| Presenteeism | 19.62 | 2.25 | 15.66 | 24.57 | 24.89 | 2.94 | 19.75 | 31.38 | 5.28 | 3.66 | -1.90 | 12.46 | 0.143 |  |
| Total work productivity impairment | 21.23 | 2.53 | 16.81 | 26.81 | 26.33 | 3.16 | 20.80 | 33.31 | 5.10 | 3.99 | -2.73 | 12.92 | 0.197 |  |
| Total activity Impairment | 21.55 | 2.00 | 17.96 | 25.85 | 26.69 | 2.46 | 22.28 | 31.97 | 5.14 | 2.23 | 0.77 | 9.51 | 0.019 |  |
| No. of Physician visits in the Past 6 months | 6.32 | 0.74 | 5.02 | 7.95 | 7.03 | 0.79 | 5.63 | 8.77 | 0.71 | 0.78 | -0.83 | 2.24 | 0.366 |  |
| Visited ER in the Past 6 Months | 0.02 | 0.01 | 0.00 | 0.05 | 0.04 | 0.02 | 0.02 | 0.10 | 0.02 | 0.02 | -0.01 | 0.06 | 0.091 |  |
| Hospitalized in the Past 6 Months | 0.04 | 0.02 | 0.02 | 0.09 | 0.08 | 0.02 | 0.04 | 0.14 | 0.04 | 0.02 | 0.00 | 0.08 | 0.071 |  |
| Absenteeism cost (in thousand ¥) | 85.49 | 34.14 | 39.08 | 187.00 | 128.98 | 54.24 | 56.57 | 294.09 | 43.49 | 66.52 | -86.89 | 173.87 | 0.496 |  |
| Presenteeism cost (in thousand ¥) | 764.20 | 92.97 | 602.08 | 969.98 | 926.76 | 116.06 | 725.06 | 1184.57 | 162.56 | 147.02 | -125.59 | 450.71 | 0.264 |  |
| Indirect cost (in thousand ¥) | 832.15 | 104.82 | 650.10 | 1065.18 | 961.74 | 122.25 | 749.65 | 1233.83 | 129.58 | 158.86 | -181.78 | 440.94 | 0.412 |  |
| Direct cost (in thousand ¥) | 604.82 | 272.86 | 249.82 | 1464.31 | 1851.47 | 858.18 | 746.41 | 4592.59 | 1246.64 | 768.29 | -259.18 | 2752.47 | 0.017 |  |
| SE: Standard Error, LCL: Lower Confidence Limit, UCL: Upper Confidence Limit | | | | |  |  |  |  |  |  |  |  |  |  |

**Table S9 Pairwise comparison of outcome variables among the caregiver groups**

| **Patients** | | **Caregiver of Schizophrenia** | | **Caregiver of Depression** | | **Caregiver of Alzheimer's disease/ dementia** | | **Caregiver of Stroke** | | **p-value^a^** | | | | | |
| --- | --- | --- | --- | --- | --- | --- | --- | --- | --- | --- | --- | --- | --- | --- | --- |
|  |  | **(1)** | | **(2)** | | **(3)** | | **(4)** | | **(1) vs. (2)** | **(1) vs.  (3)** | **(1) vs. (4)** | **(2) vs. (3)** | **(2) vs.(4)** | **(3) vs. (4)** |
| **Continuous Variable** | | **N** | **Mean ± SD** | **N** | **Mean ± SD** | **N** | **Mean ± SD** | **N** | **Mean ± SD** |  |  |  |  |  |  |
| Health-related Quality of Life | Mental Component Summary (MCS) | 126 | 44.05 ± 11.27 | 146 | 41.51 ± 10.68 | 1594 | 46.94 ± 10.37 | 342 | 46.08 ± 10.79 | 0.058 | 0.003 | 0.075 | < 0.001 | < 0.001 | 0.168 |
|  | Physical Component Summary (PCS) | 126 | 49.18 ± 8.50 | 146 | 49.55 ± 7.21 | 1594 | 51.52 ± 6.24 | 342 | 51.61 ± 6.67 | 0.698 | < 0.001 | 0.001 | < 0.001 | 0.002 | 0.809 |
|  | EQ-5D | 126 | 0.78 ± 0.16 | 146 | 0.74 ± 0.19 | 1594 | 0.82 ± 0.16 | 342 | 0.82 ± 0.16 | 0.131 | 0.003 | 0.008 | < 0.001 | < 0.001 | 0.999 |
|  | SF-6D | 126 | 0.70 ± 0.13 | 146 | 0.67 ± 0.12 | 1594 | 0.73 ± 0.13 | 342 | 0.73 ± 0.12 | 0.059 | 0.004 | 0.017 | < 0.001 | < 0.001 | 0.742 |
| Work Productivity and Activity Impairment | Absenteeism | 30 | 9.60 ± 19.51 | 54 | 10.72 ± 21.21 | 486 | 5.68 ± 16.28 | 105 | 4.07 ± 13.80 | 0.812 | 0.207 | 0.082 | 0.014 | 0.018 | 0.345 |
|  | Presenteeism | 31 | 37.10 ± 33.59 | 59 | 28.14 ± 25.43 | 501 | 25.09 ± 26.19 | 109 | 24.68 ± 26.86 | 0.159 | 0.015 | 0.034 | 0.639 | 0.418 | 0.883 |
|  | Total Work Productivity Impairment | 30 | 39.13 ± 35.63 | 54 | 34.81 ± 30.30 | 480 | 26.83 ± 27.92 | 104 | 25.84 ± 28.44 | 0.559 | 0.022 | 0.035 | 0.049 | 0.068 | 0.742 |
|  | Total Activity Impairment | 126 | 33.97 ± 28.98 | 146 | 34.32 ± 27.09 | 1594 | 24.13 ± 26.09 | 342 | 25.91 ± 28.38 | 0.919 | < 0.001 | 0.007 | < 0.001 | 0.003 | 0.262 |
| Healthcare Resource Utilization | No. of Physician Visits in the Past 6 Months | 126 | 8.44 ± 8.87 | 146 | 8.01 ± 10.14 | 1594 | 7.44 ± 9.98 | 342 | 6.33 ± 8.78 | 0.707 | 0.275 | 0.022 | 0.515 | 0.066 | 0.056 |
|  | No. of ER Visits in the Past 6 Months | 126 | 0.17 ± 0.73 | 146 | 0.64 ± 3.43 | 1594 | 0.22 ± 3.23 | 342 | 0.15 ± 1.10 | 0.132 | 0.842 | 0.846 | 0.141 | 0.018 | 0.660 |
|  | No. of Hospitalizations in the Past 6 Months | 126 | 0.50 ± 2.30 | 146 | 0.84 ± 4.48 | 1594 | 0.67 ± 6.73 | 342 | 0.67 ± 6.69 | 0.449 | 0.774 | 0.784 | 0.775 | 0.780 | 0.987 |
| Cost | Absenteeism Cost (thousand yen) | 30 | 338 ± 620 | 54 | 431 ± 926 | 486 | 206 ± 593 | 105 | 155 ± 615 | 0.626 | 0.238 | 0.154 | 0.014 | 0.027 | 0.428 |
|  | Presenteeism Cost (thousand yen) | 31 | 1397 ± 1366 | 59 | 1005 ± 986 | 501 | 939 ± 1034 | 109 | 926 ± 1067 | 0.121 | 0.019 | 0.044 | 0.639 | 0.636 | 0.905 |
|  | Indirect Cost (thousand yen) | 30 | 1460 ± 1433 | 54 | 1261 ± 1232 | 480 | 1003 ± 1098 | 104 | 955 ± 1109 | 0.505 | 0.030 | 0.042 | 0.106 | 0.115 | 0.691 |
|  | Direct Cost (thousand yen) | 126 | 1239 ± 5025 | 146 | 1989 ± 9902 | 1594 | 1601 ± 14712 | 342 | 1562 ± 14481 | 0.443 | 0.784 | 0.807 | 0.755 | 0.745 | 0.965 |
| Caregiver Reaction Assessment | Impact on Health | 126 | 2.96 ± 0.73 | 146 | 2.97 ± 0.69 | 1594 | 2.78 ± 0.73 | 342 | 2.81 ± 0.76 | 0.986 | 0.007 | 0.046 | 0.004 | 0.031 | 0.547 |
|  | Caregiver's Esteem | 126 | 2.98 ± 0.67 | 146 | 2.95 ± 0.63 | 1594 | 3.01 ± 0.62 | 342 | 3.06 ± 0.64 | 0.682 | 0.621 | 0.252 | 0.257 | 0.082 | 0.184 |
|  | Impact on Schedule | 126 | 2.71 ± 0.97 | 146 | 2.72 ± 0.91 | 1594 | 2.63 ± 0.88 | 342 | 2.63 ± 0.92 | 0.972 | 0.301 | 0.402 | 0.244 | 0.345 | 0.949 |
|  | Impact on Finances | 126 | 2.97 ± 0.80 | 146 | 2.94 ± 0.81 | 1594 | 2.73 ± 0.82 | 342 | 2.83 ± 0.91 | 0.754 | 0.002 | 0.123 | 0.003 | 0.203 | 0.047 |
|  | Lack of Family Support | 126 | 2.63 ± 0.76 | 146 | 2.56 ± 0.78 | 1594 | 2.49 ± 0.77 | 342 | 2.41 ± 0.76 | 0.506 | 0.053 | 0.007 | 0.258 | 0.042 | 0.087 |
| **Categorical Variable** | | **N** | **%** | **N** | **%** | **N** | **%** | **N** | **%** | **p-value^b^** | | | | | |
| PHQ-9 | PHQ-9 Score < 10 | 107 | 84.9% | 124 | 84.9% | 1465 | 91.9% | 305 | 89.2% | 0.998 | 0.007 | 0.208 | 0.004 | 0.187 | 0.102 |
|  | PHQ-9 Score ≥ 10 | 19 | 15.1% | 22 | 15.1% | 129 | 8.1% | 37 | 10.8% |  |  |  |  |  |  |
| a: p-value based on one-way ANOVA | | |  |  |  |  |  |  |  |  |  |  |  |  |  |
| b: p-value based on Pearson’s chi-square test | | | |  |  |  |  |  |  |  |  |  |  |  |  |
